# Avascular necrosis in pediatric systemic lupus erythematosus: a brief report and review of the literature

**DOI:** 10.1186/s12969-015-0008-x

**Published:** 2015-04-23

**Authors:** Reut Gurion, Vin Tangpricha, Eric Yow, Laura E Schanberg, Grace A McComsey, Angela Byun Robinson, for the Atherosclerosis Prevention in Pediatric Lupus Erythematosus Investigators

**Affiliations:** Division of Pediatric Infectious Diseases, Rheumatology and Global Health, Department of Pediatrics, Rainbow Babies and Children’s Hospital/Case Medical Center, Cleveland, OH USA; Division of Endocrinology, Metabolism & Lipids, Department of Medicine, Emory University School of Medicine, Atlanta, GA USA; Atlanta VA Medical Center, Decatur, Georgia USA; Duke Clinical Research Institute, Durham, North Carolina USA; Division of Pediatric Rheumatology, Duke University Medical Center, Durham, NC 27710 USA; Division of Medicine, University Hospital Case Medical Center, 1100 Euclid Avenue, Cleveland, OH 44106 USA

**Keywords:** Avascular necrosis, Systemic lupus erythematosus, Pediatrics

## Abstract

**ᅟ:**

Avascular necrosis (AVN) occurs in several chronic illnesses, including systemic lupus erythematosus (SLE), but can also occur in healthy children. There are multiple theories to explain why and how AVN occurs, but an exact mechanism has yet to be unraveled. AVN in the pediatric lupus population is understudied. The Atherosclerosis Prevention in Pediatric Lupus Erythematosus (APPLE) trial, provides an excellent venue to conduct an exploratory analysis to assess associations between AVN and demographics, SLE disease activity and vitamin D deficiency. Herein we present a brief report describing our findings, as well as reviewing the literature on AVN in SLE and other entities.

**Trial registration:**

ClinicalTrials.gov identifier: NCT00065806.

## Introduction

Proper oxygen delivery and supply is vital to the health of living tissues. Bone is no exception, and when compromise occurs, damage due to ischemic injury can ensue. Bone death by this process is called avascular necrosis (AVN). Depending on the involved joint and the extent of injury, impairment can range from minimal to bone compression and loss of function. AVN is known by many names, such as non-infectious osteonecrosis, ischemic necrosis, aseptic necrosis of bone, and subchondral avascular necrosis. AVN is not rare; in the United States it is estimated that approximately 10% of the 500,000 total joint replacements performed annually are due to AVN [1], but specific prevalence and incidence are unknown.

Clinical presentation of AVN varies, related to the size and location of the damage. Affected individuals can be asymptomatic [2], but pain is a common complaint and is often the presenting symptom [3]. Pain severity ranges and can worsen with use of the affected joint. Decreased range of motion can occur, and if an affected joint is weight bearing, abnormal gait and loss of mobility can result. AVN may be found incidentally, but often a combination of clinical history, examination and imaging such as X-rays, scintigraphy, and magnetic resonance imaging (MRI), provides the diagnosis.

AVN occurs in different conditions, including systemic lupus erythematosus (SLE), or can be idiopathic. Although pathogenesis remains unclear, several theories have risen, and multiple associations have been identified.

### Brief report

AVN is a known complication of SLE, first described in 1960 [4] by Dubois and Cozen reporting on 11 cases of AVN in 400 SLE patients. The first series addressing AVN in pediatric lupus was published in 1974 [5] with 4 cases of AVN in 10 children with lupus. We will use our report as a framework for review of the literature.

The active form of vitamin D (1,25-dihydroxy vitamin D), is known to impact both bone health and regulation of the innate immune system [6]. Cholecalciferol (vitamin D3), is produced by the skin following exposure to UVB light; ergocalciferol (vitamin D2) is the dietary supplemental form. Both are converted by the liver to form 25-hydroxy vitamin D, and then further converted in the kidney to the active form (1,25-dihydroxy vitamin D). Hypovitaminosis D is postulated to play a role in bisphosphonate-associated osteonecrosis of the jaw [7] as well as in idiopathic AVN [8-10]. Vitamin D deficiency is common in the SLE population [11] and has been associated with elevated SLE disease activity index (SLEDAI) scores [12].

### Methods

Using frozen serum and demographic data from the Atherosclerosis Prevention in Pediatric Lupus Erythematosus (APPLE) trial, we conducted an exploratory analysis to assess associations between AVN and demographics, SLE disease activity and vitamin D deficiency, defined as serum 25-hydroxyvitamin D [25(OH)D] < 20 ng/mL.

APPLE trial participants were randomized to placebo or atorvastatin 10 or 20 mg daily (depending on weight). Frozen serum collected at baseline was used to measure 25(OH)D levels by chemiluminescent assay (IDS, LTD). Quality control of the 25(OH)D measurements were assured by participation in the vitamin D external quality assessment scheme (www.deqas.org) and the NIST/NIH Vitamin D Metabolites QA Program. Those without frozen serum samples were excluded from analysis. The presence of AVN was recorded as part of the Systemic Lupus International Collaborating Clinics/American College of Rheumatology (SLICC/ACR) Damage Index and reported as an adverse event during the trial. Univariable analysis of APPLE data from baseline to 3 years was performed using chi-squared test for categorical baseline variables and Wilcoxon signed rank test for continuous variables.

### Results

Samples were available for 201/221 APPLE participants recruited from 21 sites in North America. At entry, 9/201 (0.04) had a history of AVN and another 8/192 (0.04) developed incident AVN during the study period for a total of 17 subjects included in AVN subanalyses for the current study. Among the 17 APPLE subjects with AVN, the following patient characteristics differed at baseline compared to the 184 subjects without AVN: presence of vitamin D deficiency, minority status, current therapy in a center located at a southern latitude, elevated triglycerides, and a history of hypertension and/or glomerulonephritis (Table 1). Baseline body mass index, presence of antiphospholipid antibodies, SLEDAI score, SLE disease duration and baseline corticosteroid use were not associated with AVN. Between those with a history of AVN at baseline and those with incident AVN during the APPLE study, there was no significant difference in prevalence of vitamin D deficiency, female gender, minority status, and use of steroids at entry into the study. Compared to the subjects with no past or incident AVN, subjects with AVN were more likely to have vitamin D deficiency at baseline, be non-Caucasian and have a history of hypertension, glomerulonephritis or elevated fasting triglycerides. At baseline, 4/9 subjects with a history of AVN had multifocal involvement. By the last follow up at 36 months, 8/17 subjects with AVN had developed multifocal involvement.

**Table 1:** **Univariable analysis on APPLE data at baseline to 3 years**

**Variable**	**No AVN**	**AVN**	**P-value**
	**No (%) Median (25th, 75th)**	**No (%) Median (25th, 75th)**	
Baseline 25(OH)D (ng/mL)	25.9 (18.9, 31.6)	18.7 (15.1, 32.2)	0.266
25(OH)D < 20 ng/mL	52/184 (28.3%)	9/17 (52.9%)	0.034
Minority status: non-Caucasian	113/184 (61.4%)	15/17 (88.2%)	0.028
Age (years)	15.5 (13.7, 17.6)	16.5 (14.5, 18.0)	0.207
Female	153/184 (83.2%)	14/17 (82.4%)	>0.999
SLE duration (months)	23.5 (8.0, 44.5)	25.0 (7.0, 45.0)	0.787
SLEDAI	4.0 (2.0, 6.0)	4.0 (0.0, 8.0)	0.779
History of hypertension	55/178 (30.9%)	10/17 (58.8%)	0.020
History of glomerulonephritis	58/183 (31.7%)	12/17 (70.6%)	0.001
History of nephritis/nephrosis	67/183 (36.6%)	14/17 (82.4%)	<0.001
Corticosteroid use	148/183 (80.9%)	15/17 (88.2%)	0.744
Triglycerides (mg/dL)	99.5 (74, 130.0)	145.5 (88.5, 161.0)	0.050
Total cholesterol (mg/dL)	146.0 (124.0, 173.0)	160.5 (145.5, 183.0)f	0.101
C3 (mg/dL)	99.0 (85.0, 122.0)	108.5 (99.5, 115.0)	0.175
C4 (mg/dL)	13.7 (9.0, 19.)	19.3 (15.1, 22.9)	0.017
Baseline Homocysteine (mcmol/L)	6.7 (5.6, 8.7)	6.8 (5.4, 10.0)	0.538
Latitude (°N)	40.7 (37.4, 40.9)	37.4 (36.0, 40.0)	0.004

### Brief report conclusions

This is the first report of vitamin D deficiency associated with AVN in pediatric lupus. Vitamin D deficiency was significantly associated with subjects who had or developed AVN. When we separated those who developed AVN during the 3 years of the trial, and compared median baseline levels, not just deficiency status, subjects who developed AVN had lower median vitamin D levels but this was not statistically significant likely due to low numbers. Surprisingly, we found current location in southern latitude associated with more AVN, contrary to previous reports in idiopathic AVN; however, the latitude difference between the two groups was only 3.3° with overlapping ranges, suggesting a spurious finding. Upon further evaluation of the data, it also appears that vitamin D supplementation rates varied by clinical study site; so this finding may be due to confounding by site. Associations of AVN with minority ethnicity (non-Caucasian), history of hypertension, renal involvement, and elevated triglycerides were also seen. Although there is a higher prevalence of hypovitaminosis D in patients with chronic kidney disease [13], this has not been studied specifically in SLE populations. These exploratory findings were interesting; however, due to low numbers, we were unable to perform a multivariate analysis to identify factors independently associated with AVN. In agreement with other studies [14,15], we did not find an association of AVN with antiphospholipid (aPL) antibodies. Hyperhomocysteinemia is understood to be a risk factor for atherosclerosis and atherothrombosis (endothelial damage); hyperhomocysteinemia was theorized to cause endothelial damage, leading to AVN, but the literature is inconclusive [16-19]. Our data did not show an association between hyperhomocysteinanemia and AVN. Lastly, unlike other studies, no association was seen between steroid use and AVN; however, this was only taking into account steroid use at baseline and not throughout a participant’s disease course.

The major limitation of our study is a low number of participants who developed AVN during the trial. Because of low numbers and analyzing subjects who had reported AVN at baseline, we were unable to perform a Cox hazard analysis of relative risk over 3 years. Despite no significant difference in steroid use at trial onset, our study also is limited by not having a detailed report of each patient’s previous steroid exposure. Lastly, our definition of AVN was determined by SLICC/ACR Damage Index and reported adverse events; we were unable to independently confirm the diagnosis. In addition, there were subjects who might have had asymptomatic AVN who were not diagnosed. Nevertheless, the association with hypertension and hypovitaminosis D found in our study is provocative and deserves further study.

## Review

When blood supply is interrupted, bone death is imminent. The vascular tree supplying the medulla, bone marrow, trabecular bone and endosteal portion of the cortex is complex. Some bony structures, such as the femoral head, are more vulnerable than others; their blood supply has minimal collateral circulation, and they function as weight bearing joints, thus sustaining more mechanical stress. There are several hypothesized mechanisms leading to AVN, but all share a common final pathway. Blood supply interruption leads to cellular injury, and if the affected bone is unable to repair the damage, necrosis occurs.

Many factors may contribute to AVN including: extravascular disruption (via external vascular compression due to increased fat content in the bone marrow [20-22] or bone healing [23,24], or via mechanical injury or stress [25,3,26,27]); intravascular disruption (due to thrombosis [28], embolism [22] sickle cells antibodies or immune complexes occlusion [29,30]); Vascular irritation causing spasms (theorized to be induced by vasculitis [31], radiation [32,33], and angiospasms [1]); improper angiogenesis [34]; and lastly primary osteocytes cell death [35]. It is difficult to determine which mechanism is predominantly responsible for AVN, and it is likely multiple cumulative insults in a multi-factorial process may lead to the catastrophic end result of AVN.

### AVN in pediatric SLE

**Figure 1:**
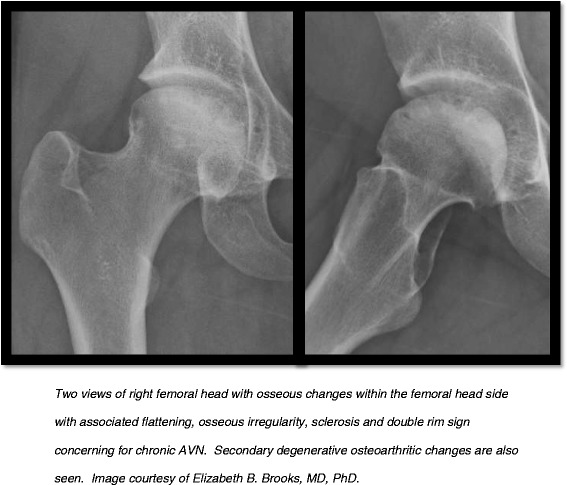
AVN in a SLE patient.

SLE has classically been associated with AVN (Figure 1) with prevalence rates in the pediatric SLE population ranging from 5-40% [5,36-38] (Table 2). The largest series by Ravelli et al. [38] (387 participants) reported a prevalence of 5.4% using data obtained from SLICC/ACR as was done in our report. Our study’s prevalence of 8.4% was slightly higher, but we fell within the reported range. In the past, a female predominance of AVN in SLE patients has been reported [5,36], but a recent large study in the adult population identified a higher percentage of male predominance in the AVN group [39]. Our data did not indicate an association with gender.

**Table 2:** **AVN in pediatric SLE population as reported by various authors**

**Author**	**Definition of AVN**	**AVN/N (%)**
Hurley et al. 1974 [5]	Radiographic findings; exact findings not defined. Performed only in symptomatic patients.	4/10 (40%)
Bergstein et al. 1974 [36]	Radiographic findings defined as: “mottling of the bone trabecular pattern, subchondral demineralization, depression or fragmentation, and irregular areas of lucency and/or sclerosis”. Performed in all patients.	14/35 (40%)
Brunner et al. 2002 [37]	No direct definition. Authors performed a retrospective chart review and calculated SLICC/ACR Damage Index score, which include AVN.	15/66 (22.7%)
Ravelli et al. 2003 [38]	No direct definition. Authors obtained information from calculated SLICC/ACR Damage Index score as well as retrospective chart review.	21/387 (5.4%)

Multiple factors have been associated with AVN in SLE, but it is steroid use that has been routinely thought of as a risk factor for developing AVN. The exact mechanism in which steroids could cause AVN has never been agreed upon, and multiple theories exist. Vascular injury due to steroid induced osteoporosis and microrotrauma was suggested [40]. Steroid induced thrombi due to hypercoagulability was proposed [41]; others theorized that steroids induced fatty liver could cause fatty emboli [42-44] leading to decreased perfusion and osteocyte death. There are also those who feel that an increased intraosseous pressure due steroid induced lipid infiltration could cause an external obstruction leading to AVN [20]. It is accepted that corticosteroids suppress angiogenesis, and it has also been postulated that this could play a role in the development of AVN [34]. However, it is important to remember that almost all patients with SLE are exposed to steroids at some point during their disease making it difficult to interpret previous findings and the true impact of steroid use on development of AVN in SLE remains unclear. In addition, one should keep in mind that there are reports of SLE patients developing AVN despite never being exposed to steroids [4,45]. In addition, patients with other rheumatic illnesses such as juvenile dermatomyositis (JDM) commonly receive significant amounts of steroids and rarely develop AVN [46]. Our data did not indicate association with current steroid use; however, we did not have the advantage of detailed steroid use information throughout the subjects’ disease course.

Our data suggested a trend towards an association between elevated triglycerides and AVN. We also found HTN, glomerulonephritis and history of nephritis or nephrosis to be associated with AVN. This association may actually point towards worse disease or increased steroid use in these individuals which could independently present a potential cause for the AVN.

### Idiopathic AVN and AVN in other childhood conditions

It is known that trauma can cause AVN, but AVN may also be non-traumatic and either idiopathic or seen in association with other medical conditions.

**Figure 2:**
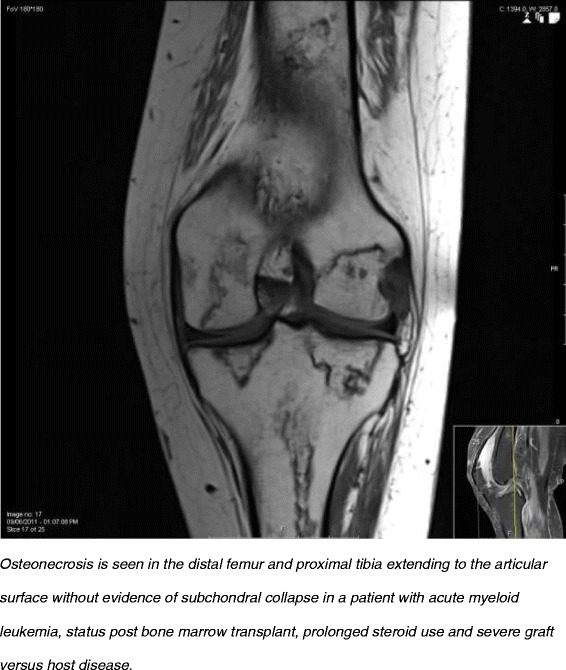
Osteonecrosis in a patient with acute myeloid leukemia.

AVN is seen in **childhood oncologic diseases**. In this entity, multifocal AVN is common [47] (Figure 2). The childhood cancer survivor study (CCSS) is a retrospective cohort with prospective follow up of children diagnosed with cancer between 1970–1986; as part of their second follow up, patients were given a questionnaire where they were asked about diagnosis of AVN [47]. This identified transplanted patients with acute lymphoblastic leukemia (ALL), acute myeloid leukemia (AML), and chronic myelogenous leukemia as well as nontransplanted patients with ALL, AML and bone sarcoma were at a higher risk of developing AVN. Similar to AVN in SLE, the exact pathogenesis in this population has not been definitively elucidated, but several risk factors were identified including older age at cancer diagnosis [48-50], steroid use [49], stem-cell transplantation [48,51,52], and radiation exposure [32]. AVN of the femoral head and of the talus has also been reported in patients with hematologic abnormalities such as ***hemophilia*** [53-56] and sickle cell disease (SCD) (discussed later). In hemophillia, AVN is likely caused due to hemarthrosis compression or thrombosis affecting either arterial or venous vasculature.

**Figure 3:**
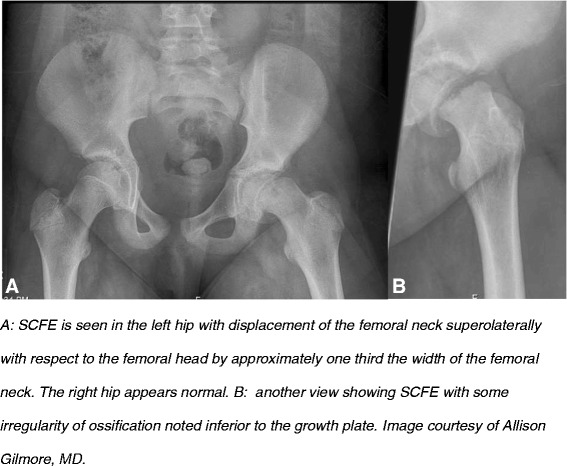
Slipped capital femoral epiphysis (SCFE).

**Slipped capital femoral epiphysis (SCFE)** is a type of idiopathic AVN that affects school age children and adolescents (Figure 3). Lehmann et al. [57] used the Kids’ Inpatient Database (KID) [58], a large publically available national database of pediatric discharges (6.70 and 7.30 million in 1997 and 2000 respectively), and found the overall incidence of SCFE in the United States to be 10.8 cases/100,000 children. Incidence was significantly higher in boys, consistent with previous reports [59]. There was a significant racial disparity with higher incidence in Blacks, Hispanics, and Asian or Pacific Islanders [57]. Incidence was significantly higher in the Northeast and West, and had seasonal variation, with more SCFE diagnoses in the summer in northern latitudes (>40°) and in the winter in southern latitudes (<40°) [57]. The pathogenesis of SCFE remains unclear: collagen abnormalities [60-62], mechanical stress [63,64], and endocrine disorders such as hypothyroidism, growth hormone deficiency [65], and obesity [66], are potential causes. A small series from India showed a significant association with vitamin D deficiency [10].

**Figure 4:**
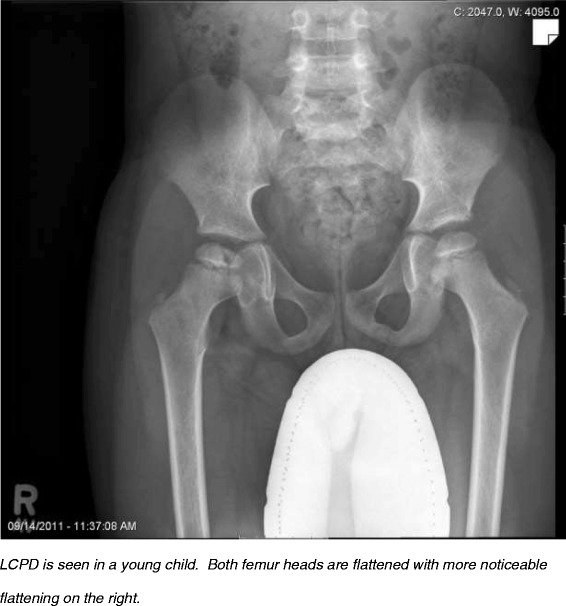
Legg-Calvé-Perthes’ disease (LCPD).

In 1910, independent descriptions of non-infectious hip pathology in the pediatric population were published by Legg [67], Calvé [68] and Perthes [69], becoming known as **Legg-Calvé-Perthes’ disease (LCPD)** (Figure 4). Konjetzny showed vascular supply interruption to the femoral head [70], and early histological analysis showed that osteonecrosis is followed by revascularization [71]. It is now understood that LCPD is caused by an ischemic injury, yet the exact etiology remains unclear. According to a meta-analysis by Perry et al. [8], this type of idiopathic AVN is common, with incidence ranging from 0.2 to 19.1 per 100,000 children less than 15 years old. There is a strong male predominance [9] and a higher incidence in Caucasians [8]. In the same meta-analysis, northern latitude was a strong predictor of LCPD even after adjustment for race. Northern latitude as a predictor of LCPD was also seen in two other UK studies [9,72].

In a large 4-decade observational study of patients with ***SCD***, an overall prevalence of 21% with AVN was reported (224/1056 patients) [73]. AVN has a higher incidence in those patients with HbSS phenotype and α-thalassemia [74]. In SCD, AVN tends to affect the femur and humerus most commonly. The natural history of this morbidity tends to differ in the different age groups. In younger children less than 8 years of age, remodeling can occur, very similarly to that which occurs in LCPD, while in adulthood, non-healing necrosis is more typical [29]. The exact pathogenesis in the setting of SCD is unknown but it is theorized that the recurrent sickling, increased blood viscosity and vasculature blockade cause stasis, leading to hypoxia and infarction in the affected bone [29]. Elevated hemoglobin [75] and hospitalization for bone infarction due to vaso-occlusive sickle crisis [73] were related with development of femoral head AVN. It is also speculated that due to this hyper-viscous state, increased intraosseus pressure from vascular occlusion may play a role [76]. In a histological comparison of bone biopsies from patients with SCD to those with idiopathic AVN, non-specific inflammation was seen in the former [77]. Genetic susceptibility to AVN was suggested. Several genes that have a role in bone metabolism were identified through examining associations of AVN with single nucleotide polymorphism (SNPs); Klotho (*KL*) gene participates in regulation of vitamin D, *BMP6* (Bone morphogenic protein) plays a role in bone formation and inflammation and Annexin A2 (ANXA2) gene regulates cell growth and mineralization [78].

### Vitamin D and AVN

In patients with LCPD, increased latitude was associated with increased risk of AVN, and although not studied, vitamin D deficiency was theorized to be associated with development of AVN [8,9]. A small series of patients with SCFE (n = 15), showed vitamin D deficiency in all patients with SCFE, and was significantly different than in controls [10]. In patients with SCD, genes involving regulation of vitamin D were involved in genetic susceptibility to AVN [78]. In rat models of osteonecrosis of the jaw, a combination of vitamin D deficiency and bisphosphonate treatment was associated with higher prevalence of AVN than with either variable alone [7]. Vitamin D is known to have a role both in bone health and in also in the regulation of the innate immune system. A pilot study assessing inflammatory markers in otherwise healthy individuals who had vitamin D deficiency showed that with vitamin D supplementation, interleukin 6, tumor necrosis factor and interferon alpha levels decreased significantly [6]. Hypovitaminosis D has higher prevalence in the SLE population [11], and a strong inverse correlation has been reported between vitamin D level and SLEDAI [12]. The association we found between vitamin D and AVN in our study should be explored further.

### Therapy

Therapeutic approach for treatment of AVN depends largely on the involved joint and the extent of the injury. Therapy for AVN in weight bearing joints such as the hip is targeted towards preservation of the joint and its function; in non-weight bearing joints the therapy may be less aggressive.

Both non-operative and operative therapies have a place in treatment of AVN. Immobilization has a role for those small lesions that will spontaneously heal, but is not a useful modality for extensive ischemic injury [79]. Electrical stimulation has been tried as an adjunct to other therapies with varying results [80]. Various medications have been used anecdotally with some benefit, including lipid-lowering drugs, anti coagulants, vasodilators and bisphosphonates [79].

The type of surgical therapy is based on the severity of joint damage. For early AVN, core decompression and percutaneous drilling is recommended. For AVN lesions prior to bone collapse, bone grafting and osteotomies are also a possibility. Once subchondral fracture collapse is apparent, bone grafting, hemi-resurfacing and total hip arthroplasty are options. Lastly, with severe joint deformity or acetabular involvement a total hip arthroplasty is indicated [79].

Stem cell treatment of femoral head AVN has been reported as useful therapy in pre-clinical disease; however, this therapeutic approach has not been standardized and will need to be studied further [81].

## Conclusions

AVN can cause significant morbidity. No single etiology is known to cause the interruption of blood supply which is the common pathway for all AVN; rather, AVN is likely the end result of a multifactorial process. There are multiple conditions associated with AVN and multiple risk factors have been identified. Through these associations and risk factors further studies may isolate potential pathways responsible for the development of AVN and suggest more effective therapies.

In the present study, we identified intriguing associations with vitamin D deficiency, elevated triglycerides, HTN, and glomerulonephritis suggesting future avenues of study and new possible approaches for AVN prevention.

## Abbreviations

ALL: Acute lymphoblastic leukemia
AML: Acute myeloid leukemia
APPLE: Atherosclerosis Prevention in Pediatric Lupus Erythematosus
AVN: Avascular necrosis
ANXA2: Annexin A2
BMP6: Bone morphogenic protein
CCSS: Childhood cancer survivor study
KID: Kids’ Inpatient Database
KL: Klotho
LCPD: Legg-Calvé-Perthes’ disease
MRI: Magnetic resonance imaging
SCD: Sickle cell disease
SCFE: Slipped capital femoral epiphysis
SLE: Systemic lupus erythematosus
SLEDAI: Systemic lupus erythematosus disease activity index
SLICC/ACR: Systemic Lupus International Collaborating Clinics/American College of Rheumatology
SNPs: Single nucleotide polymorphism
25(OH)D: 25-hydroxyvitamin
